# Multifocal Recurrent Osteomyelitis and Hemophagocytic Lymphohistiocytosis in a Boy with Partial Dominant IFN-γR1 Deficiency: Case Report and Review of the Literature

**DOI:** 10.3389/fped.2017.00075

**Published:** 2017-05-03

**Authors:** Aidé Tamara Staines-Boone, Caroline Deswarte, Edna Venegas Montoya, Luz María Sánchez-Sánchez, Jorge Alberto García Campos, Teodoro Muñiz-Ronquillo, Jacinta Bustamante, Francisco J. Espinosa-Rosales, Saul Oswaldo Lugo Reyes

**Affiliations:** ^1^Immunology Department, UMAE 25 IMSS, Monterrey, Mexico; ^2^Laboratory of Human Genetics of Infectious Diseases, Necker Branch, Institut Imagine, Paris, France; ^3^Pediatrics Department, UMAE 25 IMSS, Monterrey, Mexico; ^4^Immunodeficiencies Research Unit, National Institute of Pediatrics, Mexico City, Mexico; ^5^Infectious Disease Department, UMAE 25 IMSS, Monterrey, Mexico; ^6^Oncology Department, UMAE 25 IMSS, Monterrey, Mexico

**Keywords:** interferon gamma receptor 1 deficiency, Mendelian susceptibility to mycobacterial disease, bacille Calmette–Guérin vaccine, osteomyelitis, hemophagocytic lymphohistiocytosis, human recombinant interferon gamma, macrophage activation syndrome, multiorgan dysfunction

## Abstract

Mutations in the genes coding for cytokines, receptors, second messengers, and transcription factors of interferon gamma (IFN-γ) immunity cause Mendelian susceptibility to mycobacterial disease (MSMD). We report the case of a 7-year-old male patient with partial dominant (PD) IFN-γ receptor 1 deficiency who had suffered from multifocal osteomyelitis attributable to bacille Calmette–Guérin vaccination since the age of 18 months. He developed hemophagocytic lymphohistiocytosis (HLH), a hyper-inflammatory complication, and died with multiorgan dysfunction, despite having been diagnosed and treated relatively early. Patients with PD IFN-γR1 deficiency usually have good prognosis and might respond to human recombinant subcutaneous IFN-γ. Several monogenic congenital defects have been linked to HLH, a catastrophic “cytokine storm” that is usually ascribed to lymphocyte dysfunction and thought to be triggered by interferon gamma. This is the sixth patient with both MSMD and HLH of whom we are aware. The fact that patients with macrophages that cannot respond to IFN-γ still develop HLH, bring these assumptions into question.

## Introduction

Primary immunodeficiencies (PIDs) are a group of more than 350 rare congenital diseases with increased susceptibility to infection, autoimmunity, malignancy, allergy, and hyperinflammation. Patients with a subgroup of PID defects, known as Mendelian susceptibility to mycobacterial disease (MSMD), carry mutations in one of several genes coding for cytokines, receptors, second messengers, and transcription factors of interferon gamma (IFN-γ) immunity, maintained by interacting T-lymphocytes and macrophages (1, 2). Said mutations render those patients vulnerable to mycobacteria, mainly adverse reactions to the *bacille Calmette–Guérin* (BCG) vaccine, and environmental low-pathogenic mycobacteria (EM), but also to *Mycobacterium tuberculosis*, salmonella, and a few other intra-macrophagic bacteria (2, 3).

Hemophagocytic lymphohistiocytosis (HLH), also known as macrophage activating syndrome, is a hyperinflammatory condition resulting from any of various disorders that affect macrophages, lymphocytes, and/or natural killer cells. The pathophysiology is described as a systemic “cytokine storm” of IL-1, TNFα, IFN-γ, and IL-6, among others, with “rogue” unleashed macrophages engulfing hematopoietic cells (4). HLH is characterized by high-grade fever, lymphadenopathy, splenomegaly (with or without hepatomegaly), and cytopenias; with hyperferritinemia, hypertriglyceridemia, and hypofibrinogenemia. Here, we present the case of a 7-year-old boy who died of HLH after having been stable for 3 years, under treatment for partial dominant (PD) IFN-γR1 deficiency. The association of these two rare conditions, MSMD and HLH, has only been recognized recently; we include a review of five other cases found in the literature (Table 1).

**Table 1:** **Clinical and genetic features of six patients with MSMD and HLH**.

	P1	P2	P3	P4	P5^a^	P6
Sex	Female	Female	Male	Female	Male	Male
Age	2 months	4 years	8 years	16 years	7 years	8 years
Origin	Portugal	China	Mexico	Mexico	Mexico	Mexico
Gene	*IFNGR2*	*IFNGR1*	*IL12RB1*	*IL12RB1*	*STAT1-GOF*	*IFNGR1*
	Homozygous	Homozygous	Homozygous	Homozygous	Homozygous	Heterozygous
Exon	2		3			
Mutation	c.74_216del	c.655G>A p.G219R	c.182A>G	r.700_701ins	c.208C>T p.R70C	818del4
				TTGGTTTG		
				GTTCTGAT		
				TGCAG		
Onset	2 months	4 years	5 years	14 years	5 years	18 months
Presentation	Impetiginized eczema, HLH	Fever, mediastinal mass, HLH	Recurrent ankle abscess	Systemic lupus erythematosus	Intestinal and lymph node TB	Multifocal recurrent osteomyelitis
Infections	CMV	Pulmonary tuberculosis, EBV, MTB	Lymphadenitis, pneumonia, meningitis	Pneumonia, retinal granuloma, osteomyelitis, meningitis	Sepsis	Osteomyelitis, sepsis, pneumonia
Isolate	*Mycobacterium bovis*	MTB	*Mycobacterium gordonae*	*M. bovis*	MTB	*Staphylococcus aureus*
Status	Dead	Dead	Alive	Alive	Alive	Dead
Reference	JACI 2014	JACI 14	JoCI 2016	JoCI 2016	JoCI 2016	This report

*^a^Patient 5 from Mexico was later diagnosed with STAT1-GOF, not MSMD*.

*CMV, cytomegalovirus; EBV, Epstein–Barr virus; HLH, hemophagocytic lymphohistiocytosis; MTB, Mycobacterium tuberculosis*.

## Case Report

A 4-year-old boy was referred to our care in 2010, for a history of multifocal osteomyelitis. The patient was born in Denver, CO, USA, from Mexican non-consanguineous parents, and lived in Veracruz, Mexico. He was immunized with the BCG vaccine 1 month after birth, with no apparent adverse reactions. At the age of 18 months, he first started with persistent high fever (around 39°C) and difficulty standing up.

**Figure 1:**
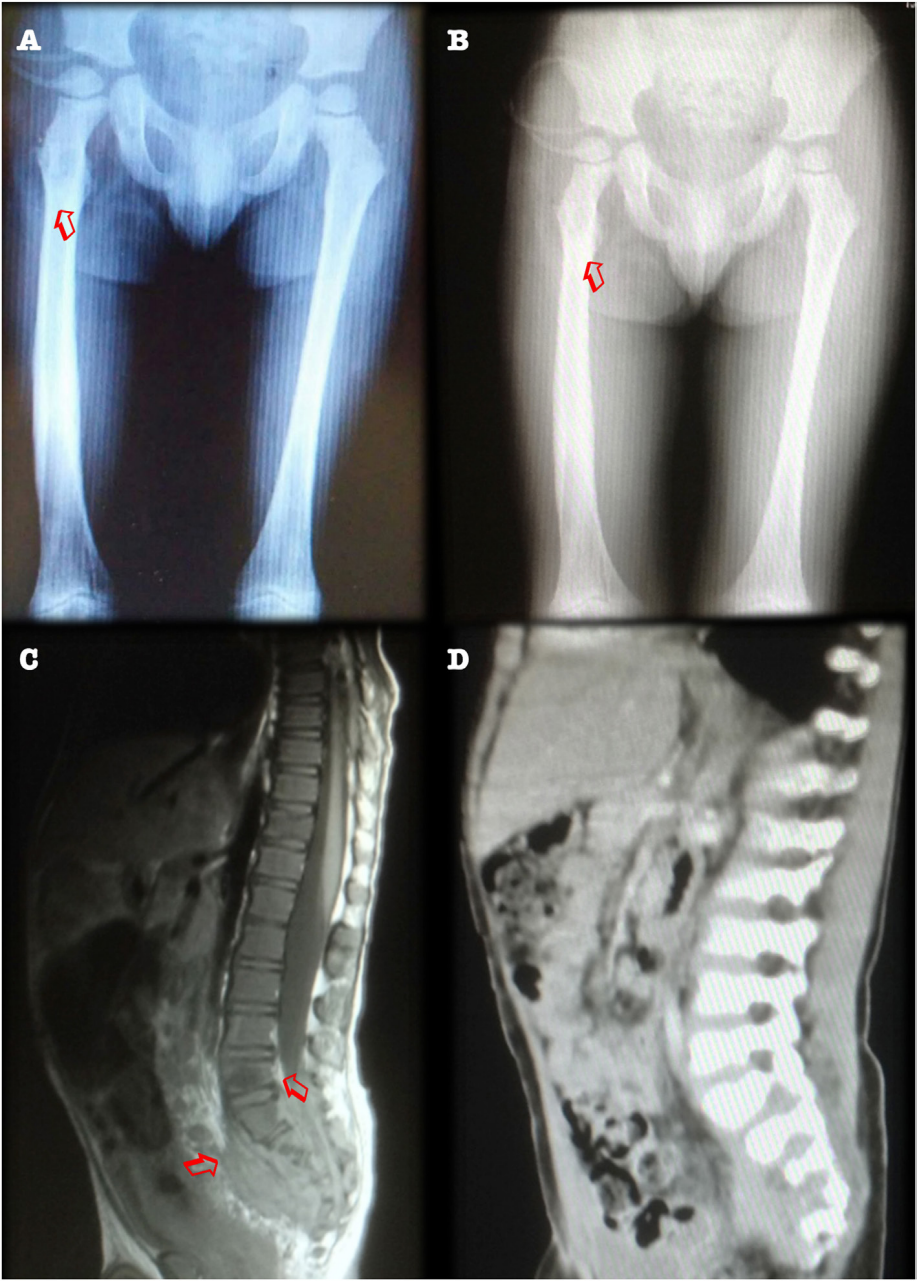
**(A)** Anteroposterior X-ray film revealing metaphyseal osteolytic lesions in femurs (arrowheads). Contrast with panel **(B)**. Radiological improvement after 1 year of antimycobacterial drug treatment, with femoral remodeling. **(C)** CT scan, sagittal view, showing presacral mass and osteolytic lesions of the S3–S4 vertebral bodies (arrowheads). Contrast with panel **(D)**. Improvement after 1 year of treatment, with vertebral remodeling and decreased antesacral mass.

On physical examination, there was limited motion of the legs and left shoulder. X-rays revealed numerous osteolytic lesions over the right femur, left tibia, frontal bone, and right clavicle (Figure 1A). Three bone biopsies obtained purulent material, although no isolate was recovered. A fourth (femoral) biopsy confirmed chronic osteomyelitis and grew *Staphylococcus aureus*, for which he was started on intravenous antibiotics (vancomycin) for 4 weeks, with partial improvement.

The initial laboratory work-up reported mild anemia, leukocytosis with neutrophilia, and thrombocytosis. IgG 1,592 mg/dl, IgM 163, IgA 237 mg/dl, IgE 43 IU/ml (within normal range for age). C3 122 mg/dl, C4 26 mg/dl. The nitro-blue tetrazolium reduction assay was normal at 45% (local lab lower limit: 40%).

An abdominal ultrasound showed hepatomegaly. A computed tomography scan from neck to pelvis found bilateral cervical, supraclavicular, and axillar lymphadenopathies, as well as a 5.5 × 4.5 cm presacral mass with lytic features, involving S2 and S3 (Figure 1C). A biopsy specimen obtained during exploratory laparotomy was described as granulomatous, after which he was started on antimycobacterial antibiotics.

**Figure 2:**
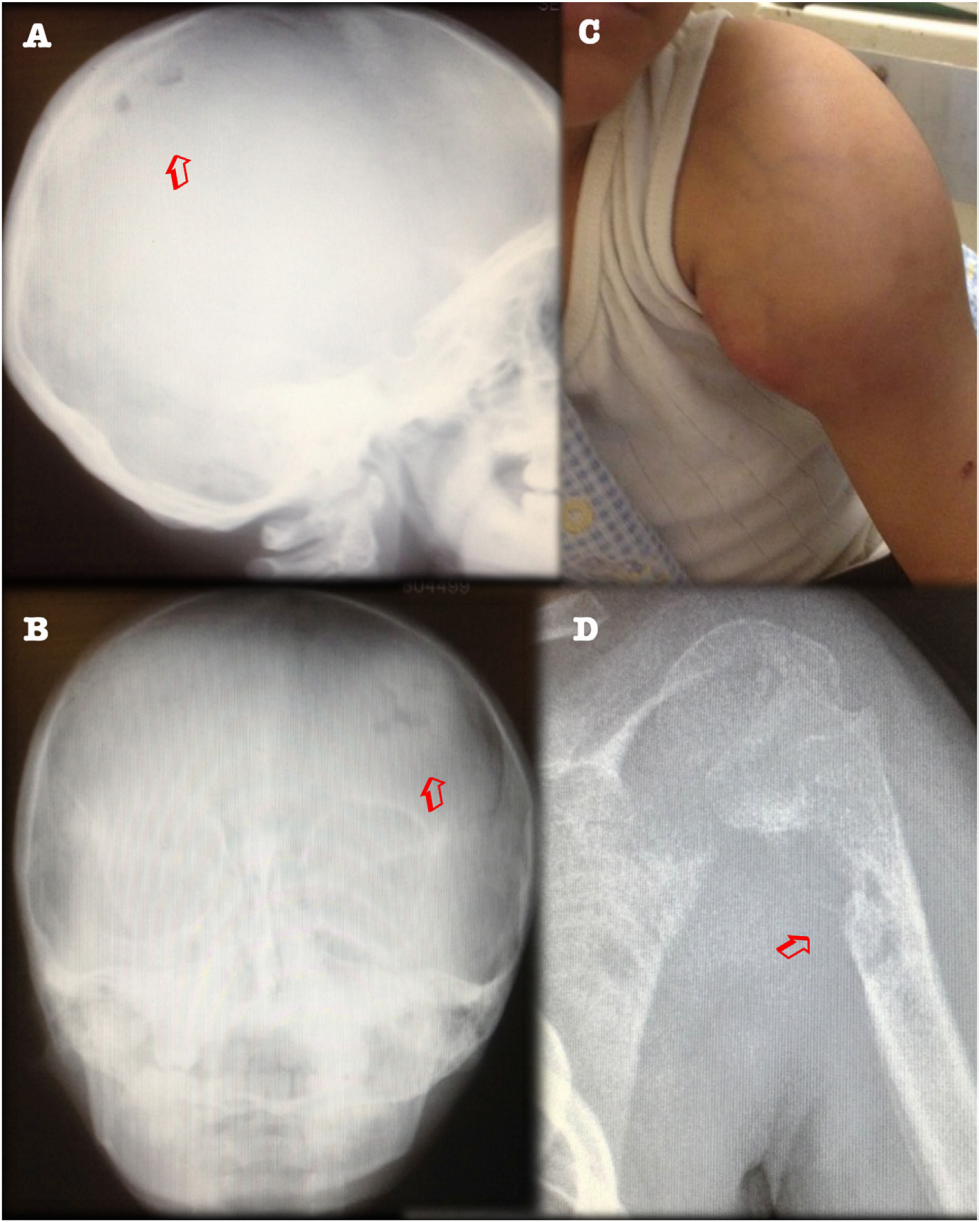
**Clinical relapse with upper arm abscess, and new lytic lesions in parietal bone (left panels) and left humerus (right panels)**. **(A)** X-ray of skull, lateral view. **(B)** Posteroanterior view, showing osteolytic lesions (arrows). **(C)** Swelling of left shoulder over abscess. **(D)** X-ray of left humerus showing osteolytic lesions (arrow).

**Figure 3:**
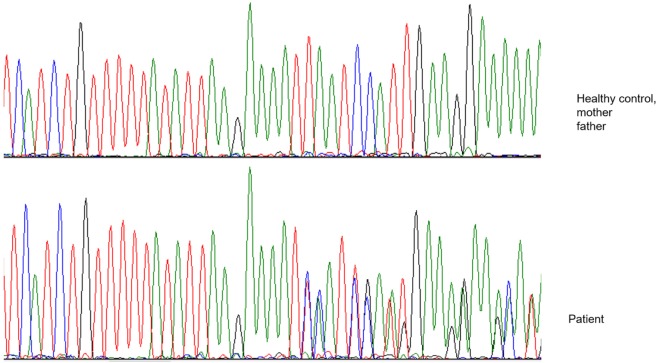
**Electropherogram showing a heterozygous four-nucleotide deletion in the patient, as contrasted with a healthy control and the patient’s parents**.

After 1 year of clinical and radiological improvement (Figures 1B,D), the multidrug regime was shifted to maintenance phase, but 6 months later the patient complained of pain in the left arm, again showing radiographic signs of osteomyelitis and abscess on physical examination (Figure 2). Genomic DNA amplification with the polymerase chain reaction and automated Sanger sequencing revealed a monoallelic small deletion in exon 6 of *IFNGR1* (818del4) (Figure 3). He was kept on high-dose anti-tuberculous antibiotics, monthly IVIG, and bi-annual IV zoledronic acid for 3 years.

**Figure 4:**
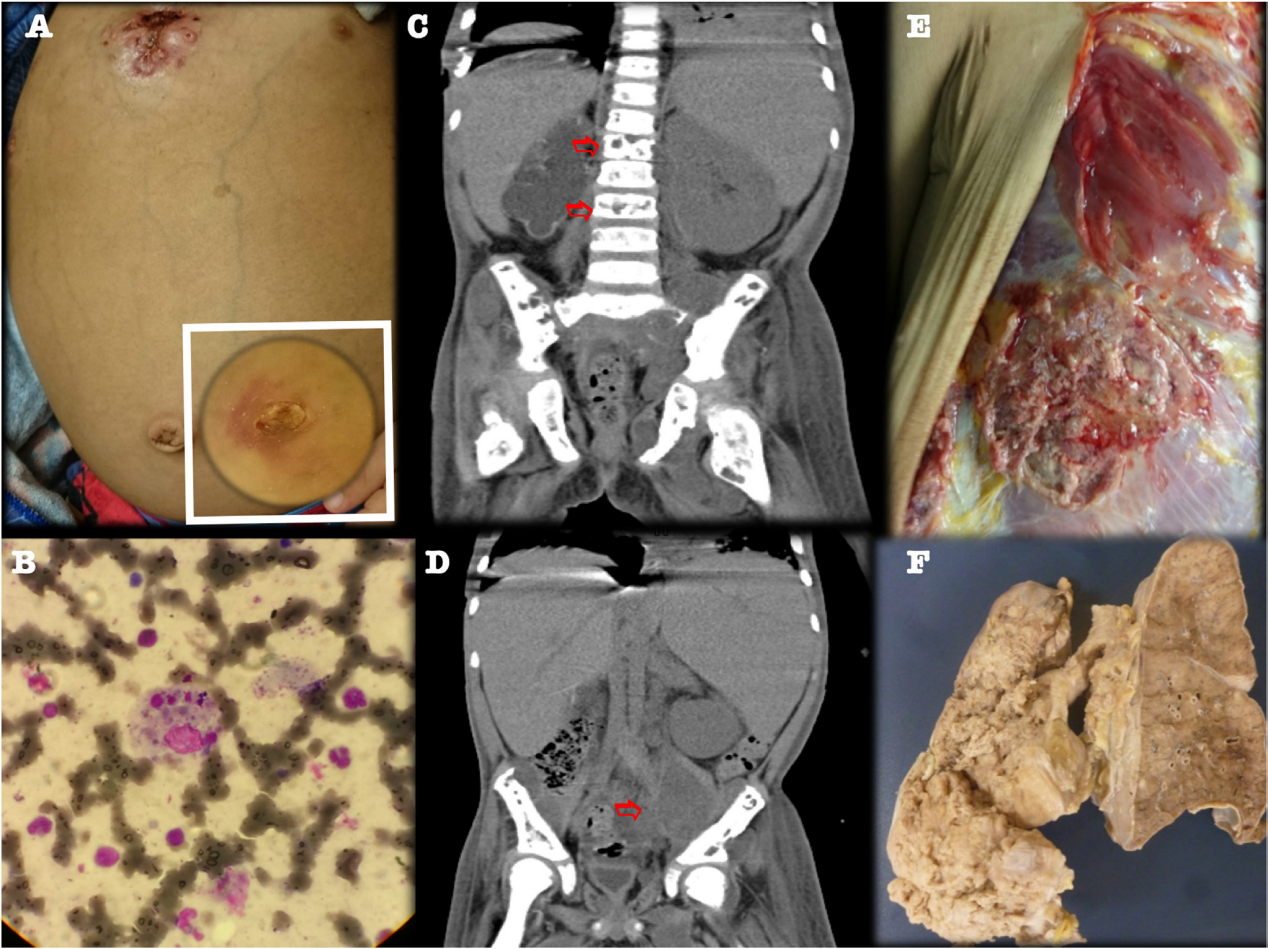
**Third relapse with CT scans showing left psoas abscess and multiple osteolytic lesions**. **(A)** Fistulized multifocal pneumonia and left psoas abscess. **(B)** Bone marrow aspirate (Hematoxylin and eosin staining, 40×) showed histiocytes phagocytizing three blood cell lineages. **(C,D)** CT scan, coronal view, showing lumbar vertebral osteolytic lesions (panel C, arrows) and left psoas abscess (panel D, arrow). **(E,F)** Gross appearance of chest wall and lung specimen at autopsy, confirming multifocal fistulized pneumonia.

At age 7, he developed high fever, massive hepato-splenomegaly, multiple lymphadenopathies, severe hemolytic anemia and lymphopenia (Hb 5 g/dl, WBC 6840, ANC 5,800, lymphocytes 700/mm^3^, platelets 213,000), with serum ferritin 5,540 μg/l, and triglycerides 366 mg/dl. A bone marrow aspirate confirmed hemophagocytosis (Figure 4; Figure S1 in Supplementary Material), and Epstein–Barr virus (EBV) serology identified high viral titers and viral load (691–5,000 copies/ml). The patient fulfilled five of eight Histiocyte Society criteria (5) for HLH (namely: fever, splenomegaly, cytopenias, hyperferritinemia, and hemophagocytosis in bone marrow). While still on a quadruple antimycobacterial regime, he received the HLH-04 chemoimmunotherapy protocol that includes etoposide, dexamethasone, and cyclosporine, with complete clinical and laboratorial recovery.

Five months later, however, he again developed fever, respiratory distress, and bone pain. Image evaluation showed new lumbar and pelvic osteolytic lesions, left psoas abscess, and multifocal pneumonia, with a fistulizing chest wall abscess, despite continued prophylactic treatment (Figure 4). The patient was started on IV broad-spectrum antibiotics, gammaglobulin, and rituximab (to treat his recurrent EBV-associated HLH), despite which his condition deteriorated progressively, with massive lung bleeding and multiorgan dysfunction, leading to irreversible cardiac arrest. An autopsy found multifocal pneumonia, fistulized chest wall abscess, numerous septic thrombi, and soft tissue abscesses.

## Discussion

We report the case of a school-age boy who suffered from multiple recurrent osteomyelitis and systemic lymphadenopathy due to mycobacterial infection. Despite an early diagnosis, after initial improvement, he developed HLH and died.

Primary immunodeficiency was suspected in this patient because of the early, recurrent, unusual infections. The diagnosis of MSMD was pursued once it was clear that the osteomyelitis, abscesses, and granulomata had been caused by mycobacteria. *IFNGR1* was a strong contender for genetic etiology, as the severe clinical presentation of multifocal osteomyelitis following BCG vaccination has been consistently reported in the context of IFN-γR deficiency (2, 6–8). The PD genotype correlates with a less severe clinical presentation than the autosomal recessive complete deficiency (2). The mutation found in this patient, 818del4, is in a hotspot for small deletions (4). The IFN-γ receptor chain lacks its intracytoplasmic recycling domain, and the truncated non-functional IFN-γR1proteins accumulate at the cell surface (8), interfering with the function of the wild-type receptors. Patients with PD IFN-γR deficiency are usually treated with prolonged systemic antibiotic therapy (four to five oral anti-tuberculosis drug regime) (2), and they might respond well to high doses of human recombinant IFN-γ (50 μg/m^2^/dose, up to 200 μg/m^2^/dose, three times a week for 2–3 years), before considering a gradual decrease of both IFN-γ and anti-tuberculosis drugs, according to clinical and imaging improvement. Hematopoietic stem cell transplantation is not generally indicated.

Hemophagocytic lymphohistiocytosis has been increasingly reported as an infectious (viral or bacterial) complication in PID defects, including chronic granulomatous disease (X-linked and autosomal recessive), severe-combined immunodeficiency, Wiskott–Aldrich syndrome, DiGeorge anomaly, X-linked agammaglobulinemia, Hyper-IgD syndrome, ectodermal dysplasia with immunodeficiency, autoimmune lymphoproliferative syndrome (FAS-ALPS), cyclic neutropenia, tumor necrosis factor-1 receptor associated periodic syndrome (TRAPS), familial Mediterranean fever, NLRC4; and thus far five patients with defects in the IFN-γ circuit (9, 10). In addition, several monogenic PID diseases have been linked to HLH (11), including the four known genes causing familial HLH (*PRF1, UNC13D, STX11, STXBP2*), and other defects considered as predisposing to HLH: the partial albinism syndromes with an “accelerated phase”: Griscelli syndrome type 2 (*RAB27A*), Chediak Higashi syndrome (*LYST*), the Hermansky Pudlak syndrome type 2 (*AP3B1*); and the X-linked lymphoproliferative diseases (both SAP and XIAP deficiencies, caused by *SH2D1A* and *BIRC4* mutations, respectively). These defects affect either lytic granules traffic or antiapoptotic proteins in lymphocytes (natural killer/cytotoxic cells). Finally, a few autoimmune diseases may develop HLH as the macrophage activation syndrome: systemic lupus erythematosus, juvenile arthritis.

The pathogenic mechanism of HLH is not clear. It is generally considered a T-cell disorder of impaired activation (11), to depend on activated NK cells (9), and to be driven by elevated IFN-γ, TNFα, and IL-6 (4). However, IFN-γ receptors are located on macrophages (and elsewhere). If macrophages are unresponsive, as they are in *IFNGR* mutations, how can they overreact to the point of becoming hemophagocytic histiocytes? From mouse models, we know that HLH can also be induced by excess IL-4 (12) or by TLR9 overstimulation (13). TLR9s are intracellular receptors, present in macrophages and other innate immune cells, that recognize CpG motifs in DNA sequences. Mycobacteria are strong inducers of toll-like receptors (TLR2, 4, and 9) (14). We can only speculate as to how in these patients, chronic mycobacterial infection might overstimulate macrophages through TLR9 (10). A small but significant proportion of patients with PID have mutations in more than one gene. To rule out a bigenic disease in this patient, we need to perform whole-exome sequencing in the proband and his parents.

Implications for clinical practice of this case include to delay the administration of the BCG vaccine in apparently healthy newborns without risk factors and to suspect IFN-γR1 deficiency in patients with multiple osteomyelitis. For newborns with a family history of MSMD, the BCG vaccine is formally contraindicated. And, secondary (infectious) HLH, also known as the macrophage activation syndrome, must be suspected early and treated aggressively in patients with PID.

## Ethics Statement

The author subscribes the 1964 Declaration of Helsinki for Medical research involving human subjects. The study of this patient was preceded by informed consent from the patient’s family. His and his family identity are protected. Case reports are exempt from our Institutional Review Board approval on account of the study design.

## Author Contributions

ATSB clinically diagnosed and treated the patient, conceived of the report, and reviewed and approved the final version for publication. CD: data analysis and interpretation, critical review, and final approval for publication. JB: data analysis and interpretation, critical revision and final approval of the version to be published. LS-S: data collection, clinical care, critical review, and final approval. JC and TR: clinical care, data collection, critical review, and final approval. EM: clinical care, data collection, figure editing, fact checking, critical review, and final approval. FE-R: critical review and final approval. SR: conception of the work, drafting the article, and discussion.

## Conflict of Interest Statement

The authors declare that the research was conducted in the absence of any commercial or financial relationships that could be construed as a potential conflict of interest.

## Supplementary Material

Figure S1 **Bone marrow aspirate showed histiocytes phagocytizing three blood cell lineages**. Hematoxylin and eosin staining, 40×.

## Abbreviations

BCG, bacille Calmette–Guérin; HLH, hemophagocytic lymphohistiocytosis; MSMD, Mendelian susceptibility to mycobacterial disease; IFNG, interferon gamma; PID, primary immunodeficiency.
