# Supplementary figures and images for: Multifocal Recurrent Osteomyelitis and Hemophagocytic Lymphohistiocytosis in a Boy with Partial Dominant IFN-γR1 Deficiency: Case Report and Review of the Literature
Source: Front Pediatr. 2017 May 3;5:75. doi: 10.3389/fped.2017.00075 (PMC5413492; doi:10.3389/fped.2017.00075)

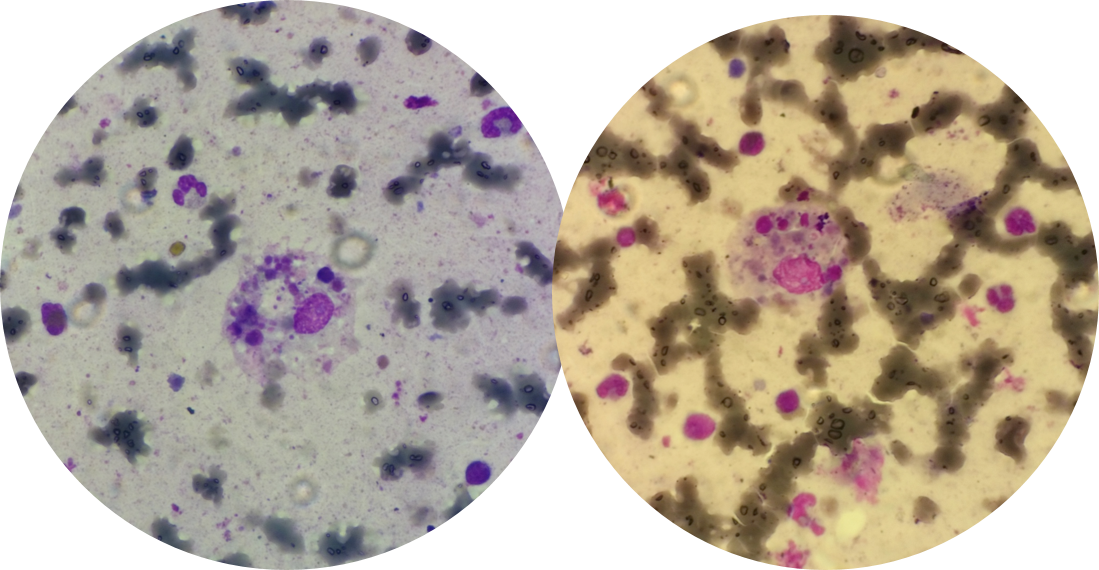

Supplement: Figure S1 — Bone marrow aspirate showed histiocytes phagocytizing three blood cell lineages. Hematoxylin and eosin staining, 40×. [file Image_1.TIF]
